# A Validated Phenotyping Algorithm for Genetic Association Studies in Age-related Macular Degeneration

**DOI:** 10.1038/srep12875

**Published:** 2015-08-10

**Authors:** Joseph M. Simonett, Mahsa A. Sohrab, Jennifer Pacheco, Loren L. Armstrong, Margarita Rzhetskaya, Maureen Smith, M. Geoffrey Hayes, Amani A. Fawzi

**Affiliations:** 1 Department of Ophthalmology, Northwestern University Feinberg School of Medicine, Chicago, IL 60611; 2 Center for Genetic Medicine, Northwestern University Feinberg School of Medicine, Chicago, IL 60611; 3 Division of Endocrinology, Metabolism, and Molecular Medicine, Department of Medicine, Northwestern University Feinberg School of Medicine, Chicago, IL 60611; 4 Department of Anthropology, Northwestern University, Evanston, IL; 5 Northwestern Comprehensive Center on Obesity, Northwestern University Feinberg School of Medicine, Chicago, IL 60611

## Abstract

Age-related macular degeneration (AMD), a multifactorial, neurodegenerative disease, is a leading cause of vision loss. With the rapid advancement of DNA sequencing technologies, many AMD-associated genetic polymorphisms have been identified. Currently, the most time consuming steps of these studies are patient recruitment and phenotyping. In this study, we describe the development of an automated algorithm to identify neovascular (wet) AMD, non-neovascular (dry) AMD and control subjects using electronic medical record (EMR)-based criteria. Positive predictive value (91.7%) and negative predictive value (97.5%) were calculated using expert chart review as the gold standard to assess algorithm performance. We applied the algorithm to an EMR-linked DNA bio-repository to study previously identified AMD-associated single nucleotide polymorphisms (SNPs), using case/control status determined by the algorithm. Risk alleles of three SNPs, rs1061170 (*CFH*), rs1410996 (*CFH*), and rs10490924 (*ARMS2*) were found to be significantly associated with the AMD case/control status as defined by the algorithm. With the rapid growth of EMR-linked DNA biorepositories, patient selection algorithms can greatly increase the efficiency of genetic association study. We have found that stepwise validation of such an algorithm can result in reliable cohort selection and, when coupled within an EMR-linked DNA biorepository, replicates previously published AMD-associated SNPs.

Age-related macular degeneration (AMD) is a multifactorial neurodegenerative disease that is the leading cause of blindness in western individuals over the age of 65^1^,^2^,^3^,^4^. Clinical presentation of AMD is heterogeneous, with many genetic and environmental risk factors contributing to its pathogenesis^5^. The rate of identification of AMD-associated genetic risk factors, including but not limited to single nucleotide polymorphisms (SNPs) in *CFH*, *ARMS2* and *HTRA1* genes, has increased rapidly with the utilization of genome-wide association studies (GWAS)^6^,^7^,^8^,^9^. These studies have led to a better understanding of AMD pathophysiology, creation of genetic based prediction models and a plethora of AMD pharmacogenomics studies^8^,^10^,^11^,^12^,^13^,^14^,^15^. GWAS studies have also identified environmental exposures that interact with AMD genetic risk factors, highlighting the importance of developing accurate criteria for clinical phenotyping in order to discriminate disease and control populations^16^,^17^. One important barrier to genetic association studies is the time consuming process of patient recruitment, phenotyping, and DNA collection necessary to build sufficiently powered cohorts. This process can be accelerated by implementing electronic medical record (EMR)-linked DNA bio-repositories, which allow multiple unrelated fields of research to share a large, common pool of genetic data coupled to a searchable EMR, significantly facilitating phenotype-genotype comparisons^18^,^19^,^20^,^21^,^22^. The use of high-throughput clinical phenotyping (HTCP) algorithms that apply specific inclusion and exclusion criteria to clinical data, available through the EMR, could generate a large cohort of potentially eligible study subjects and, in the case of EMR-linked DNA bio-repositories, would capitalize on previously genotyped or imputed data^23^,^24^. As the number and size of EMR-linked DNA bio-repositories grow, the need for accurate, validated HTCP algorithms continues to increase^25^.

HTCP algorithms have been used in ophthalmologic research; a recent systematic review identified seven North American studies that reported EMR based algorithms to identify patients with a diagnosis of uveitis^26^. All of the identified studies used inclusion criteria based on international classification of disease-9 (ICD-9) codes and three of these seven studies described validation of their algorithm. These studies investigated the epidemiology and treatment response of uveitis, but not genetic associations. Another study investigating ocular complications after anti-vascular endothelial growth factor therapy employed an algorithm to identify exudative AMD, which included any patient with ICD-9 diagnosis codes 364.00, 364.05, 364.10, 364.11, however this study did not describe a validation procedure for their algorithm^27^.

Despite the rapid growth of SNP association studies in AMD populations, validated HTCP algorithms have yet to be implemented in the field. We hypothesized that a patient cohort identified with an HTCP algorithm from an EMR-linked DNA bio-repository can be used to perform a SNP association study using previously acquired DNA samples to replicate published AMD associations. To test our hypothesis, we developed an HTCP algorithm to identify AMD and control patients using EMR data, which we validated through expert chart review. We then applied our HTCP algorithm to an institutional EMR-linked bio-repository to test our hypothesis.

## Methods

### Ethics

The study was approved by the Institutional Review Board at Northwestern University and adhered to the tenets set forth by the Declaration of Helsinki; informed consent was obtained from patients prior to enrollment in this study.

### Algorithm Development

**Figure 1:**
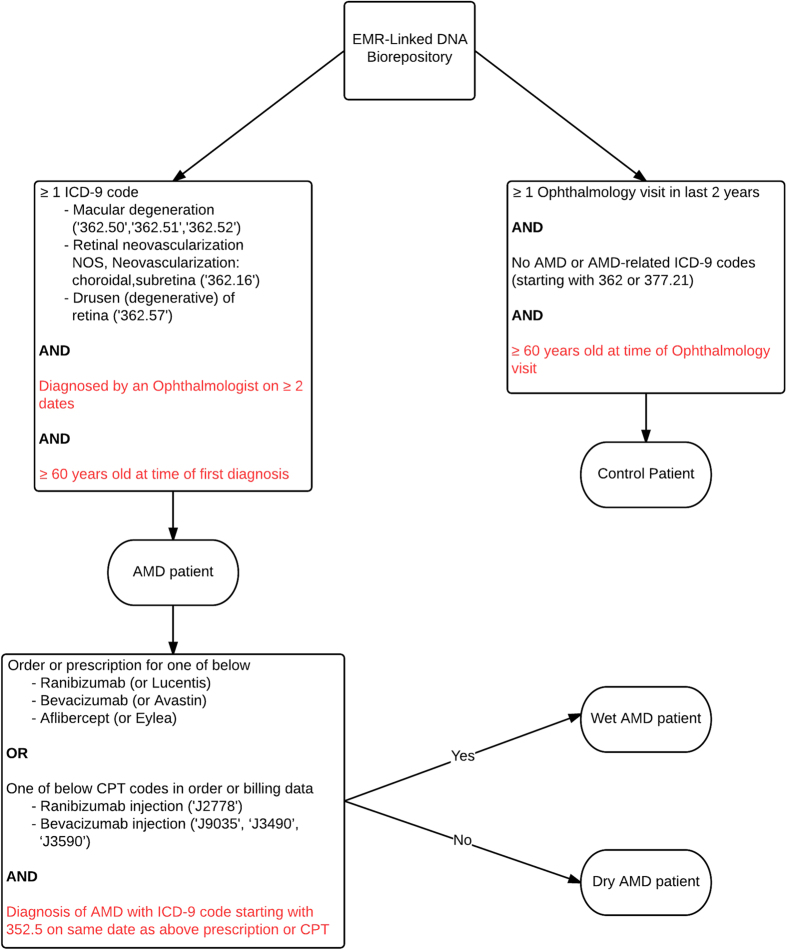
High-throughput clinical phenotyping algorithm outline. Final HTCP algorithm applied to EMR-linked DNA biorepository. Red criteria were added after first round of case selection/expert chart review. ICD-9: International Classification of Disease-9, CPT: current procedural terminology.

We developed the algorithm to identify all AMD cases using the criterion of AMD ICD-9 codes entered by an ophthalmologist (362.50, 362.51, 362.52, 362.16, 362.57). To classify cases as “wet” AMD cases within this population, we additionally required a current procedural terminology (CPT) code (J2778: ranibizumab injection, J9035, J3490 or J3590: bevacizumab injection), or an order or prescription for ranibizumab , bevacizumab, or aflibercept. This initial algorithm was tested by unsupervised random selection of 20 suspected AMD patient charts (10 dry and 10 wet cases of AMD) from the Northwestern University Department of Ophthalmology. Based on this initlal pilot study, we revised the HTCP algorithm to require subjects to be ≥60 years of age at the time of the first AMD diagnosis and to have ≥2 visits that were associated with the AMD ICD-9 codes. Furthermore, an ICD-9 code starting with 362.5 on the same date as the procedural CPT code or medication order was required for “wet” AMD classification. All AMD cases not meeting the wet AMD criteria were labeled as “dry” AMD (Fig. 1). Patients were classified as controls if they had ≥1 ophthalmology visit within the last two years, were ≥60 years of age at the time of the visit, and did not receive an AMD or AMD-associated diagnosis (we excluded the following non-specific or unrelated ICD-9 codes 362 or 377.21).

The modified HTCP algorithm was re-tested on the Northwestern University clinical EMR, and was set up to extract a selection of 100 charts: 30 dry AMD, 30 wet AMD and 40 control charts. None of the 20 charts from the original pilot study were included in this validating study. The annonymized charts selected by the algorithm were verified by experienced graders/retina specialists (MAS, AAF) who examined the clinical notes and retinal imaging (retinal fundus photographs, angiography and optical coherence tomography) of these 100 charts. A case was considered to have a diagnosis of “wet” AMD if at least one eye was determined to have wet AMD pathology by the graders. The positive predictive value (PPV), negative predictive value (NPV), and false negative rate (FNR) for overall AMD diagnosis and “wet vs dry” sub-classification were calculated to ensure we have achieved our preset criterion for HTCP having >90% accuracy. Once validated, the HTCP algorithm was then applied to all 11,075 subjects enrolled in the NUGene Project, an EMR-linked DNA bio-repository at Northwestern University Center for Genetic Medicine, to identify AMD cases and controls for the subsequent genetic association study. We specifically ensured that none of the patients/charts used in the validation or pilot studies were participants in the NUGene Project.

### Genotyping

We initially selected 11 SNPs based on previous studies showing significant association with AMD: rs1061170, rs1410996 (*CFH*), rs10490924 (*ARMS2*), rs11200638 (*HTRA1*), rs2230199 (*C3*), rs833069 (*VEGFA*), rs8017304 (*RAD51L1*), rs4151667, rs541862, rs641153 (*CFB)*, rs9332739 (*C2)*^10^,^11^,^28^,^29^,^30^,^31^,^32^. Through prior investigations, as part of the Electronic Medical Records and Genomics (eMERGE) network, genome-wide genotype data, imputed to >36 million SNPs using the 1000 Genomes Project cosmopolitan reference panel with IMPUTE2 were available for 38 of the 61 AMD cases and 167 of the 332 controls subjects identified with the HTCP algorithm^21^,^33^,^34^. For AMD case subjects that did not have available genotype data, we performed direct genotyping of 7 of the 11 SNPs (7 SNPs at *CFH, ARMS2, HTRA1, C3, VEGFA* and *RAD51L1*). Selection of these SNPs was based on a *P* value threshold of <0.19 from allele case/control associations using the imputed genotype data from the initial 38 cases and 167 controls. SNPs were genotyped directly from bio-banked genomic DNA samples that were previously isolated from peripheral blood leukocytes with the Gentra Autopure system at the Northwestern University Center for Genetic Medicine Genomics Core Facility. SNP genotyping was performed by direct sequencing of PCR products using either the forward or reverse primer used as sequencing primer (NEB Q5® High-Fidelity DNA Polymerase following manufacturer’s recommended protocols) at the Northwestern University Center for Genetic Medicine Genomics Core Facility. Primers and T_m_ are listed in Supplementary Table 1. We excluded genetic data from non-European-ancestry patients (4 out of 61 AMD cases and 25 out of 167 controls). None of the final 57 AMD cases had been included in the prior pilot or validation studies.

### Statistical Analysis

Demographic and past medical history data was compared between algorithm identified cases and controls and analyzed using Chi-squared and student-t tests. For genotyped SNPs, a logistic regression between AMD case-control status and allelic dosage was performed using PLINK to estimate the odds ratios (OR) and 95% confidence intervals to assess the significant association for each SNP with the diagnosis of AMD^35^. Regression models assumed an additive genetic model and adjusted for age, sex, body mass index (BMI), and smoking history, which are known confounders of AMD^36^. Similarly, we used the frequentist approach in SNPTEST v.2.2.0 for imputed SNPs to perform the same regression analyses^37^. SNP allele counts from both imputed and genotyped cases were used together to calculate final allelic ORs and *P* values. We considered SNP associations to be significant if they met a Bonferroni multiple comparison corrected *P* value threshold of *P* < 0.05/11 SNPs ~ 4.5 × 10^−3^.

## Results

### Algorithm development

Of the 20 suspected AMD patient charts selected by the initial algorithm, 45% (9/20) of patients were correctly classified as either dry or wet AMD. In the dry AMD category, misclassified cases included pattern dystrophy, diabetic retinopathy and central serous chorioretinopathy. In the wet AMD category, misclassified cases included proliferative diabetic retinopathy, high myopia with lacquer crack, idiopathic macular scar, end-stage retinopathy of prematurity, and birdshot chorioretinopathy.

After additional modifications (≥2 diagnosis dates, ≥60 years of age at diagnosis and clinic visit, and requiring AMD diagnosis at the time of CPT codes and medication orders) to improve the algorithm, the readers performed revalidation of the algorithm using the clinical EMR database. Of the 60 patients classified by the algorithm as AMD and 40 classified as controls, we found overall 94% (94/100) of patients were correctly classified as either AMD or control. Of the 60 patients identified as having a diagnosis of AMD, 5 were misclassified, including a case of proliferative diabetic retinopathy, a case of posterior vitreous detachment with recurrent vitreous hemorrhage, a case of atypical angioid streaks, a case of pattern dystrophy, and a case of ruptured macroaneurysm. Of the 40 patients identified as controls, 1 case was misclassified and found to have a large macular scar with history of polypoidal choroidal vasculopathy. For the overall AMD classification, the PPV was 91.67% (55/60), NPV was 97.50% (39/40), and FNR was 1.79% (1/56) (Table 1).

We further evaluated the algorithm performance in terms of AMD sub-classification (wet vs dry AMD). Of the 55 correctly classified AMD cases, 26 were identified as dry and 29 as wet AMD by the algorithm. Four of the 26 cases identified as dry AMD were found to be wet AMD following expert review. Three of the 29 cases identified as wet AMD were found to be dry AMD following expert review. PPV, NPV and FNR of wet and dry determination were calculated (Table 1).

### Algorithm Application to EMR-linked DNA bio-repository to confirm established AMD SNP associations

The refined HTCP algorithm identified 61 AMD cases and 332 controls within the NUGene project bio-repository. Of these, 38 AMD cases and 167 controls had genome-wide imputed data available. All SNPs tested imputed well (IMPUTE2 info score ≥0.75). The remaining 23 AMD cases were genotyped for the 7 SNPs of interest. Low quality sequencing occurred at rs1410996 in one AMD case, which was excluded from the rs1410996 allele association analysis. No additional controls were genotyped. Following exclusion of non European-ancestry subjects, a total of 57 AMD cases (imputed + directly sequenced) and 142 controls remained.

Demographics and past medical history, including diagnosis of type 2 diabetes mellitus, glaucoma and cataracts, of the AMD cases and controls used in SNP analysis are shown in Table 2. Age at last eye exam, gender, smoking history and BMI data was available for all AMD cases and controls. AMD cases were significantly older (average age 78.3 vs 69.3), had a significantly higher rate of cataracts (86.9% vs 68.9%), and trended towards having a lower BMI than controls.

The risk alleles of 3 previously identified AMD associated SNPs, rs1061170 (*CFH*), rs1410996 (*CFH*), and rs10490924 (*ARMS2*), were significantly associated (*P* value < 4.5 × 10^−3^) with AMD case/control status as identified by the HTCP (Table 3). rs11200638 (*HTRA1*) and rs2230199 (*C3*) approached significance but did not meet the Bonferroni corrected *P* value threshold. Significant allele associations were in the same direction and had similar ORs to previous reports. rs833069 (*VEGFA*) and rs8017304 (*RAD51L1*), as well as the SNPs for which direct genotyping was not obtained in the additional cases, were not significantly associated with algorithm-identified AMD cases/control status in our study.

## Discussion

Recent advances in genotyping and sequencing technology have significantly outpaced the development of HTCP phenotyping capabilities, causing labor-intensive patient identification and DNA collection to be the rate-limiting step in genetic association studies^38^. As the use of EMR-linked DNA bio-repositories expands, improved HTCP algorithms for cohort selection offers an appealing alternative to automate these processes and share clinically linked genotype data across research fields. These methods are of particular importance for chronic, complex diseases like AMD that are associated with a large number of genetic and environmental risk factors. HTCP algorithms will require a multi-step validation method that achieves sufficiently high phenotyping accuracy, in particular high PPV, necessary for identifying genetic variants that are associated with multifactorial diseases.

We found that relying on ICD-9 codes alone for AMD patient selection was not satisfactory. The addition of ICD-9-linked CPT codes or medication records and age restrictions improved algorithm accuracy at identifying both AMD (wet and dry) and control patients. 61 of 11,075 subjects enrolled in the EMR-linked DNA bio-repository were assigned AMD case status by the HTCP algorithm (0.55%), expectedly lower than the prevalence reported in the age 50 and older population as this database contained subjects of all ages. Demonstrating associations with SNPs previously shown to be associated with AMD, with odds ratios and confidence intervals that substantially overlap with those from the literature, argues that the PPV and NPV achieved by this algorithm are high enough to properly identify AMD cases and controls. The PPV for both dry and wet AMD subtype determination by the algorithm was <90% (73.3% and 86.7%, respectively) and the FNRs were significantly higher than the FNR for overall AMD case determination (12.0% and 16.1% vs 1.8%). The relative weakness of the algorithm in discriminating AMD subtypes is likely multifactorial and complicated by the spectrum of AMD retinal pathology. Further improvements could include the addition of searchable keywords within the EMR free text in addition to ICD9/CPT codes. Application to a larger AMD cohort will be needed to determine if the algorithm can be reliably used to differentiate wet and dry AMD.

Three of the seven previously reported AMD-associated SNPs examined in this study were found to be significantly associated (at Bonferroni correction threshold) with case/control status identified by the HTCP algorithm, and one of the SNPs (rs833069) trended in the opposite direction from that previously published. Potential explanations for why more SNPs were not significantly associated with case/control status include inadequate power due to sample size or insufficient phenotyping accuracy. Additionally, genetic heterogeneity between the previous populations used to identify the SNP associations and the NUGene project population may partially account for these findings. Increased compatibility between EMR systems will allow for more wide spread application of HTCP algorithms and will greatly increase the potential sample size.

One barrier to widespread use of HTCP algorithms is the lack of standardization across EMRs. Although the HTCP algorithm was sufficiently accurate in this study to identify some genetic associations, accuracy will become more difficult when algorithms are applied across multiple EMR systems, especially when they attempt to include complex additional criteria such as treatment response. Although ICD-9 and CPT codes are shared across US EMR systems, validation of this HTCP algorithm on external EMRs will be important in assessing its performance in other EMR systems. Shared billing codes and increased utilization of EMR organization tools continue to improve, including efforts to develop external informatics infrastructure on which to normalize EMR data^39^. Particularly, for the purpose of addressing Meaningful Use standards, phenotyping algorithms have been successfully applied to clinical EMR platforms and accurately identified specific patient cohorts^40^,^41^. Additionally, introduction of active learning to HTCP algorithms has been shown to decrease the number of clinical data annotations necessary to achieve a precise classification model^42^. Similar approaches in modifying EMR-linked DNA bio-repositories and HTCP algorithms are possible and would likely improve the classification function.

Multiple AMD associated SNPs, including those confirmed in this study, are believed to play an important role in the progression through specific AMD stages^43^. Utilizing combinations of genetic, clinical and demographic data, several AMD progression risk prediction models have been validated in independent patient cohorts^44^,^45^,^46^,^47^. Accuracy of modeling the risk for progression from early stage AMD to advanced stages of either geographic atrophy (GA) or choroidal neovascularization (CNV) improves with the inclusion of relevant genetic markers (GA: C-statistic = 0.94, CNV: C-statistic = 0.96) compared to phenotype-only models (C-statistic = 0.63 to 0.89)^12^. Additionally, AMD risk prediction models that incorporate a higher number of AMD associated SNPs within the *CFH* gene, compared to models that use only one or two, have been shown to be more accurate^12^. Therefore, great insight can be gained by exploring the conferred risk of haplotype combinations, even when the association between individual haplotypes and AMD are already known. Utilization of rapid association studies and validated HTCP algorithms may be an ideal method for identifying and confirming additional SNPs for this purpose.

Results from pharmaco-genomic studies on the effect of AMD-associated SNPs on clinical response to anti-VEGF treatment have been conflicting^14^,^15^,^48^. Expansion of an AMD HTCP algorithm to include treatment response has the potential to strengthen these pharmaco-genomic studies by accessing larger treatment cohorts. Identification of eye specific AMD status rather than individual AMD status will be important when exploring pharmacogenomics and treatment response in future studies; ICD-9 and CPT codes are currently not linked to a specific eye. Future use of ICD-10, which includes left and right eye specific diagnostic codes, may facilitate eye level HTCP classification.

The American Academy of Ophthalmology (AAO) 2012 recommendations for genetic testing discussed the value of genetic testing for multifactorial disorders such as AMD^49^. They recommended avoiding routine genetic screening until a specific treatment or surveillance is proven to be beneficial to patients with a given genotype. Significant advances need to be made in AMD research, including those discussed above, before personal sequencing data can directly influence patient care^50^. We believe that validated HTCP algorithms, such as the one developed in this study, when combined with EMR-linked DNA bio-repositories will become valuable tools to increase the efficiency of association studies and should be used to bring us closer to the ultimate goal of personalized medicine in AMD treatment.

## Additional Information

**How to cite this article**: Simonett, J. M. *et al.* A Validated Phenotyping Algorithm for Genetic Association Studies in Age-related Macular Degeneration. *Sci. Rep.* **5**, 12875; doi: 10.1038/srep12875 (2015).

## Supplementary Material

Supplementary Table 1

## Figures and Tables

**Table 1:** Final Algorithm Performance Metrics.

Classification	PPV	NPV	FNR
Overall AMD	91.7%	97.5%	1.8%
Dry AMD	73.3%	95.7%	12.0%
Wet AMD	86.7%	92.9%	16.1%

Final algorithm performance metrics for classifying cases as AMD, regardless of dry vs wet status, and for determining dry vs wet status of identified AMD cases. PPV: Positive predictive value, NPV: negative predictive value, FNR: false negative rate.

**Table 2:** Demographic Characteristics of HTCP Defined AMD cases and controls.

Phenotype	AMD identified cases	Control identified cases	*P*
Age at last eye exam	78.3	69.3	**5.9 × 10^−16^**
Female sex	70.5%	78.4%	0.15
History of smoking	52.5%	52.1%	0.95
BMI	26.3	27.9	0.07
Type 2 DM	29.5%	34.1%	0.62
Glaucoma	42.6%	35.5%	0.36
Cataracts	86.9%	68.9%	**5.3 × 10**^**−3**^

BMI: Body mass index, DM: Diabetes mellitus. Bolded *P* values are statistically significant (*P *<* 0.05*).

**Table 3:** Pooled Imputed and Directly Genotyped Association Results Between Previously Identified AMD Risk Alleles and HTCP Defined AMD case/control status.

SNP	Near by gene	Previously reported risk allele	RAF in AMD Cases	RAF in controls	OR (CI)	*P*	Previously reported OR (CI)	Previously reported RAF	Published source
rs1061170	*CFH*	C	0.580	0.363	2.43 (1.55–3.79)	**2.3E-04**	1.86 (1.77–1.97)	0.49	Sofat *et al.,* 2012
rs10490924	*ARMS2*	T	0.321	0.201	1.88 (1.15–3.07)	**2.0E-03**	2.76 (2.72–2.80)	0.30	Fritsche *et al.,* 2013
rs1410996	*CFH*	C	0.732	0.584	1.94 (1.20–3.14)	**2.6E-03**	1.98 (1.44–2.72)	0.60	Mori *et al.*, 2007
rs11200638	*HTRA1*	A	0.304	0.205	1.69 (1.03–2.78)	9.6E-03	1.80 (1.34–2.39)	0.31	Hadley *et al.*, 2010
rs2230199	*C3*	C	0.607	0.448	1.90 (1.22–2.97)	0.033	1.42 (1.37–1.47)	0.20	Fritsche *et al.*, 2013
rs8017304	*RAD51L1*	A	0.670	0.581	1.46 (0.92–2.31)	0.13	1.11 (1.08–1.14)	0.61	Fritsche *et al.*, 2013
rs833069	*VEGFA*	G	0.286	0.377	0.66 (0.41–1.06)	0.04	1.69 (1.26–2.26)	0.26	Galan *et al.*, 2010

SNP: Single nucleotide polymorphism, RAF: Risk allele frequency, OR (CI): Odds ratio (95% Confidence Interval). Bolded *P* values are statistically significant after Bonferroni correction (*P* < 4.5 × 10^−3^).
