# Supplementary material for: A Validated Phenotyping Algorithm for Genetic Association Studies in Age-related Macular Degeneration
Source: Sci Rep. 2015 Aug 10;5:12875. doi: 10.1038/srep12875 (PMC4530462; doi:10.1038/srep12875)
Supplement: Supplementary Table 1 [file srep12875-s1.doc]

**SREP-15-06564A Supplementary Tables**

**A Validated Phenotyping Algorithm for Genetic Association Studies in Age-related Macular Degeneration**

Joseph M. Simonett, Mahsa A. Sohrab, Jennifer Pacheco, Loren L. Armstrong, Margarita Rzhetskaya, Maureen Smith, M. Geoffrey Hayes, PhD, Amani A. Fawzi

Supplementary Table 1: Genotyping primers

| SNP | Gene | Chromosome | Position | Sequence (5'->3') | T_m_ |
| --- | --- | --- | --- | --- | --- |
| rs1061170 | *CFH* | 1 | 196690107 | CAGAAATAGGGCCAAGAAAAGAGT | 66 |
|  |  |  |  | ATGTAACTGTGGTCTGCGC |  |
| rs11200638 | *HTRA1* | 10 | 122461028 | GGCTCTCTGCGAATACGGAC | 66 |
|  |  |  |  | CGCGTCCTTCAAACTAATGGA |  |
| rs2230199 | *C3* | 19 | 6718376 | CCCTCGCACCTCCTTCAC | 69 |
|  |  |  |  | CCTCTGGCTGGCACCTCAAT |  |
| rs1410996 | *CFH* | 1 | 196727803 | TCAGGACCCAGGAAAACTTTAGG | 67 |
|  |  |  |  | AGGCACTGAGAAGCAAAGAAAC |  |
| rs833069 | *VEGFA* | 6 | 43774842 | AAACTCGCGGGAAGGAAGAC | 66 |
|  |  |  |  | TCCTAGGTGTTGGGGGAAGT |  |
| rs10490924 | *ARMS2* | 10 | 122454932 | GATGGTAACTGAGGCGGAGG | 68 |
|  |  |  |  | GGCATGTAGCAGGTGCATTG |  |
| rs8017304 | *RAD51B* | 14 | 68318360 | GGGAATCACAAGGCTCTGCT | 68 |
|  |  |  |  | ACAACCCCAGGTTCAAGGTC |  |

Sequencing primers used for subject genotyping. SNP: Single nucleotide polymorphism, T_m_: Melting temperature.
